# Clinical characteristics and thrombotic risk of atrial fibrillation with obstructive sleep apnea: results from a multi-center atrial fibrillation registry study

**DOI:** 10.1186/s12872-022-02773-9

**Published:** 2022-07-25

**Authors:** Wei Xu, Yan-min Yang, Jun Zhu, Shuang Wu, Juan Wang, Han Zhang, Xing-hui Shao, Ran Mo, Jiang-shan Tan, Jing-yang Wang

**Affiliations:** Emergency Center, National Center for Cardiovascular Disease, National Clinical Research Center of Cardiovascular Diseases, Fuwai Hospital, Chinese Academy of Medical Sciences and Peking Union Medical College, No. 167 Beilishi Road, Xicheng District, 100037 Beijing People’s Republic of China

**Keywords:** Atrial fibrillation, Non-central nervous system embolism, Sleep apnea, Stroke, Thrombosis

## Abstract

**Background:**

Sleep apnea is a risk factor for atrial fibrillation (AF) but it is underdiagnosed. Whether obstructive sleep apnea (OSA) is correlated with thrombotic risk in AF remains unclear. The aim of the present study was to analyze the clinical characteristics and assess the thrombotic risk of AF with OSA.

**Methods:**

In the present registry study,1990 consecutive patients with AF from 20 centers were enrolled. The patients were divided into 2 groups depending on whether they presented with both AF and OSA. All the patients were followed up for 1 year to evaluate the incidences of stroke and non-central nervous system (CNS) embolism.

**Results:**

Of the 1990 AF patients, 70 (3.5%) and 1920 (96.5%) patients were in the OSA group and non-OSA group, respectively. The results of the multivariate logistic model analysis showed that male sex, body mass index (BMI), smoking, and major bleeding history were independent risk factors for patients with AF and OSA. The comparison of the Kaplan–Meier curves using the log-rank test revealed that AF with OSA was correlated with an increased risk of non-CNS embolism (*p* < 0.01). After multivariate adjustments were performed, OSA remained an independent risk factor for non-CNS embolism (HR 5.42, 95% CI 1.34–22.01, *p* = 0.02), but was not correlated with the risk of stroke in patients with AF.

**Conclusions:**

The present study revealed that male sex, high BMI values, smoking, and major bleeding history were independent risk factors for patients with AF and OSA. Moreover, OSA was an independent risk factor for non-CNS embolism in AF. Our results indicate that non-CNS embolism requires focus in patients with AF and OSA.

**Supplementary Information:**

The online version contains supplementary material available at 10.1186/s12872-022-02773-9.

## Background

Atrial fibrillation (AF) is the most common clinically significant arrhythmia and affects 33.5 million individuals worldwide [1]. Due to the rapid aging of the population, AF is becoming a primary public health concern with increased mortality, high comorbidity, and rising health care costs [2]. Sleep apnea was recognized as a risk factor for AF [3]; however, its diagnostic rate was underestimated because patients find overnight testing for diagnostic confirmation is inconvenient [4, 5]. Thus, early recognition of patients with AF under high suspicion of sleep apnea and having them undergo polysomnography screening is crucial. However, few studies have been conducted on the clinical characteristics of AF combined with sleep apnea. Conversely, it remains vague whether obstructive sleep apnea (OSA) is correlated with thrombotic risk in patients with AF, and studies in this area are constrained. The objectives of the present study were to investigate the clinical characteristics and second, assess the thrombotic risk of AF with OSA.

## Methods

### Study design and population

The aim of the present multi-center prospective registry study from China was pointed to investigate the clinical characteristics and assess the 1-year outcomes of patients with AF from emergency departments. A total of 2016 consecutive patients from 20 representative centers around China from November 2008 and October 2011 were enrolled. The inclusion criteria of this registry study were as follows: patients admitted to the emergency room due to AF confirmed using an ECG and those previously diagnosed with AF and re-confirmed using an electrocardiography (ECG). Moreover, AF was classified as paroxysmal, persistent, or permanent-AF on admission [6]. Twenty-six patients were excluded as their baseline data were incomplete. A sleep apnea diagnosis had to be assessed using polysomnography. An apnea-hypopnea index (AHI) of ≥ 5 combined with symptoms, including a history of snoring, daytime sleepiness, obesity, retrognathia, or hypertension was defined as OSA. All the diagnoses were confirmed by experienced tertiary physicians. The study protocols complied with the Declaration of Helsinki and have been approved by the ethics committee of each center.

### Collection of baseline characteristics

Baseline characteristics including demographic information, vital signs on admission, medical history, and medications, were collected by interviewing the participants and physicians and reviewing the medical records. Comorbidities included myocardial infarction, coronary artery disease, congenital heart disease, valvular heart disease, heart failure, left ventricular ejection fraction (LVEF) < 45%, hypertension, diabetes mellitus, previous stroke or transient ischemic attack (TIA), left ventricular hypertrophy by echo or ECG, smoking, dementia or cognitive defects, chronic obstructive pulmonary disease (COPD), hyperthyroidism, and prior major bleeding. Data on these comorbidities were collected at admission and the diagnoses were based on clinical records by experienced physicians. Details regarding medications such as rate and rhythm control agents, anticoagulants, and antiplatelet agents were also collected in this study.

### Follow-up and outcomes

Trained researchers took 1 year ± 4 weeks to complete the follow-up through telephone interviews, outpatient service, or a delivery of the medical records. The endpoints were stroke/TIA and non-central nervous system (CNS) embolism. Stroke referred to a focal neurological deficit that lasted more than 24 h and was confirmed using imagological examination. Non-CNS embolism referred to an extracranial systemic embolic event, which was defined as vascular occlusion resulting from embolism confirmed by surgery or imaging, including limb artery thrombosis, renal artery thrombosis, mesenteric ischemia, etc.

### Statistical analysis

In the baseline data, non-normally distributed continuous variables were expressed as medians with interquartile ranges and were compared using the Mann–Whitney U test. Categorical variables were presented as frequencies and percentages and compared using the Chi-square test. Univariate and multivariate logistic regression were used to identify the clinical characteristics of AF combined with sleep apnea. Kaplan–Meier curves and log-rank tests were used to compare the two groups’ survival curves. We also created univariate cox regression models to calculate hazard ratios (HR) and 95% confidence intervals (CI) for stroke/TIA and non-CNS-embolism. Multivariate Cox regression was also conducted for stroke/TIA and non-CNS embolism, during which risk factors including sex, heart rate, heart failure, age, diabetes mellitus, hypertension, stroke/TIA, BMI, left ventricular ejection fraction (LVEF) < 45%, and smoking were adjusted for. IBM SPSS Statistics version 26.0 (IBM Corp., Armonk, NY, USA) was used for statistical analysis. All statistical tests were 2-tailed, and P-value < 0.05 was considered statistically significant.

## Results

A total of 2016 patients with AF were enrolled in the present registry study, of which 26 patients with incomplete data were excluded, and 1990 were included in the final analysis.

**Fig. 1:**
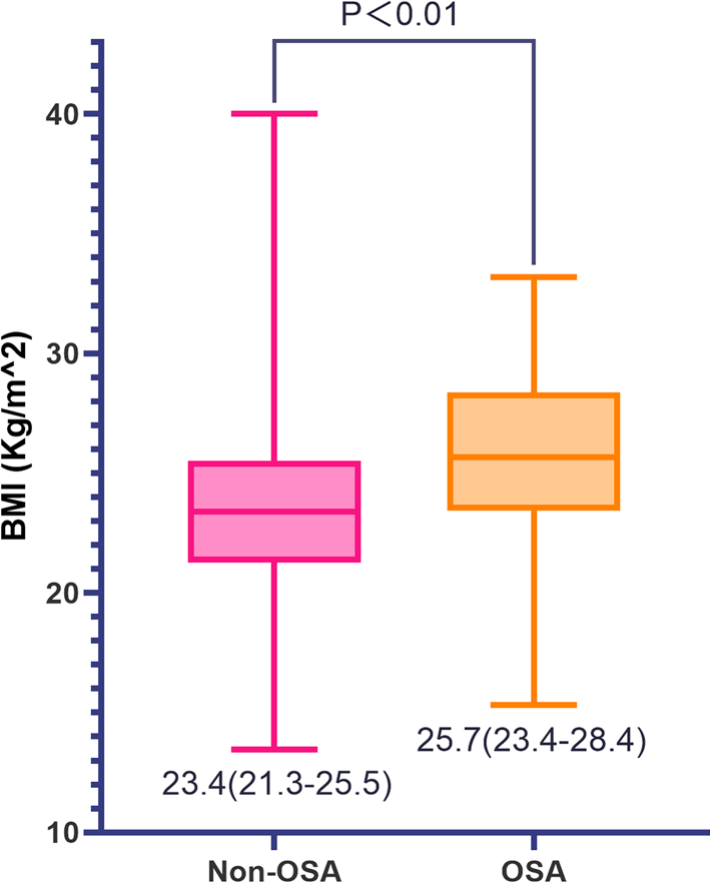
BMI values in AF patients with and without OSA. *AF* atrial fibrillation; *BMI* body mass index; *OSA* obstructive sleep apnea

The baseline characteristics and treatments in patients with AF with and without OSA are shown in Table 1. The median age was 71 years, and 897 (45.1%) of the patients in the present study were male. Notably, 70 of the patients (3.5%) had AF combined with OSA. Compared to those without sleep apnea, patients with AF and OSA were younger, more likely to be male and smokers, had higher BMIs, and tended to have histories of major bleeding. In addition, the comorbidities, including myocardial infarction, coronary artery disease, heart failure, diabetes mellitus, COPD, and dementia, did not differ significantly between these two groups. The CHA_2_DS_2_ -VASc score in the two groups were comparable. The two groups did not differ significantly in terms of medications taken. For patients who used warfarin, the INR range was comparable in the two groups (Additional file 1: Table S1). As shown in Fig. 1, the median BMI of the AF with OSA group was 25.7 kg/m^2, whereas the median BMI of the non-OSA group was 23.4 kg/m^2 (*p* < 0.01).

**Table 1:** Baseline characteristics of AF patients with and without OSA

	Total	Non-OSA	OSA	*p*-value
	n = 1990	n = 1920	n = 70	
Male (n [%])	897 [45.1%]	841[43.8%]	56 [80.0%]	< 0.01
Age (y)	71 (60–78)	71 (60–78)	66.5 (55.5–73.25)	< 0.01
BMI (Kg/m^2^)	23.4 (21.3–25.7)	23.4 (21.3–25.5)	25.7 (23.4–28.4)	< 0.01
SBP (mmHg)	130 (117–146)	130 (118–146)	127.5 (110–150)	0.86
DBP (mmHg)	80 (70–90)	80 (70–90)	80 (70–95)	0.17
Heart rate (beat/min)	97 (80–120)	97 (80–120)	98 (80–120)	0.87
Type of AF (n [%])				0.35
Paroxysmal	608 [30.6%]	584 [30.4%]	24 [34.3%]	
Persistent	449 [22.6%]	430 [22.4%]	19 [27.1%]	
Permanent	933 [46.9%]	906 [47.2%]	27 [38.6%]	
*Comorbidities*				
Myocardial infarction (n [%])	147 [7.4%]	141 [7.3%]	6 [8.6%]	0.70
Coronary artery disease (n [%])	835 [42.0%]	808 [42.1%]	27 [38.6%]	0.56
Congenital heart disease (n [%])	43 [2.2%]	43 [2.2%]	0 [0%]	0.21
Valvular heart disease (n [%])	333 [16.7%]	322 [16.8%]	11 [15.7%]	0.09
Heart failure (n [%])	744 [37.4%]	716 [37.3%]	28 [40.0%]	0.65
Left ventricular ejection fraction < 45% (n [%])	382 [19.2%]	364 [19.0%]	18 [25.7%]	0.16
Hypertension (n [%])	1110 [51.2%]	1064 [51.0%]	46 [55.7%]	0.09
Diabetes mellitus (n [%])	309 [15.5%]	296 [15.4%]	13 [18.6%]	0.48
Previous stroke or TIA (n [%])	374 [18.8%]	362 [18.9%]	12 [17.1%]	0.72
LVH by ECG or echo (n [%])	322 [16.2%]	305 [15.9%]	17 [24.3%]	0.17
Smoking (n [%])	425 [21.4%]	386 [20.1%]	39 [55.7%]	< 0.01
Dementia or cognitive defects (n [%])	44 [2.2%]	43 [2.2%]	1 [1.4%]	0.65
Emphysema or COPD (n [%])	228 [11.5%]	220 [11.5%]	8 [11.4%]	0.98
Hyperthyroidism (n [%])	66 [3.3%]	65 [3.4%]	1 [1.4%]	0.66
Prior major bleeding (n [%])	48 [2.4%]	43 [2.2%]	5 [7.1%]	0.01
CHA_2_DS_2_ -VASc score				0.01
0–1	335	312 [16.3%]	23 [32.9%]	
2–4	1051	1017 [53.0%]	34 [48.6%]	
5–9	604	591 [30.78%]	13 [18.6%]	
*Medication status*				
Diuretic(n [%])	767 [38.5%]	740 [38.5%]	27 [38.6%]	1.00
β blocker(n [%])	874 [43.9%]	845 [44.0%]	29 [41.4%]	0.67
ACEI(n [%])	458 [23.0%]	436 [22.7%]	22 [31.4%]	0.09
ARB(n [%])	318 [16.0%]	306 [15.9%]	12 [17.1%]	0.79
Calcium channel blocker(n [%])	461[23.2%]	443 [23.1%]	18 [24.7%]	0.61
Digoxin(n [%])	609 [30.6%]	592 [30.8%]	17 [24.3%]	0.24
Aspirin(n [%])	1092 [54.9%]	1058 [55.1%]	34 [48.6%]	0.28
Clopidogrel (n [%])	138 [6.9%]	132 [6.9%]	6 [8.6%]	0.58
Statin (n [%])	476 [23.9%]	463 [24.1%]	13 [18.6%]	0.56
Warfarin (n [%])	335 [16.8%]	327 [17.0%]	8 [11.4%]	0.22

*ACEI* angiotensin-converting enzyme inhibitors; *AF* atrial fibrillation; *ARB* angiotensin receptor blockers; *BMI* body mass index; *COPD* chronic obstructive pulmonary disease; *DBP* diastolic blood pressure; *ECG* electrocardiogram; *LVH* levy left ventricular hypertrophy; *OSA* obstructive sleep apnea; *SBP* systolic blood pressure

**Fig. 2:**
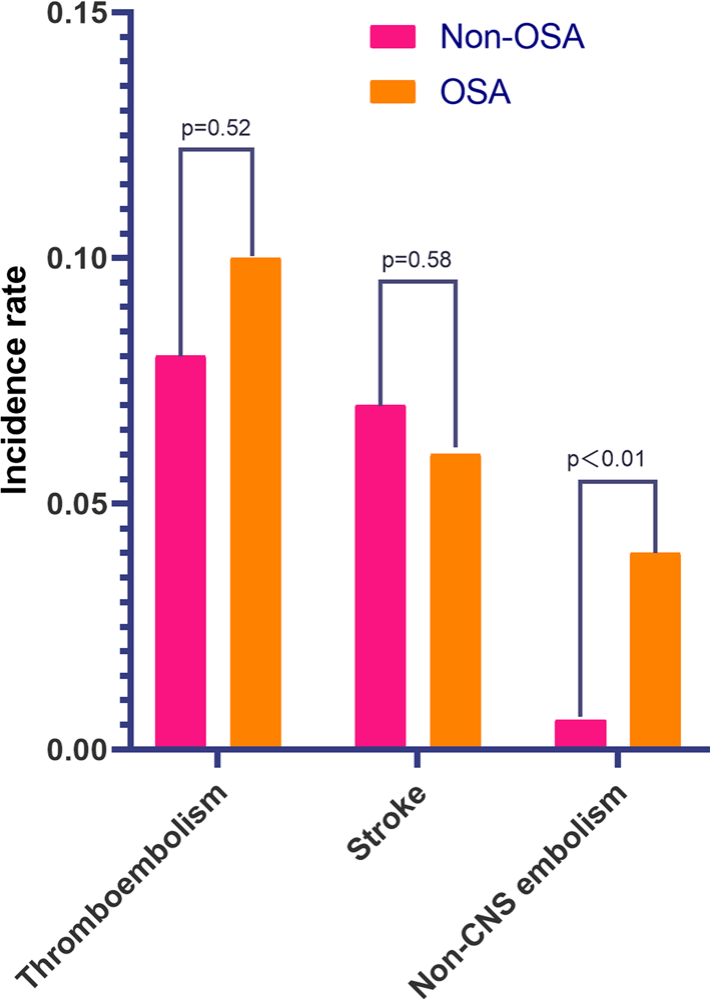
Clinical outcomes in AF patients with and without OSA. *AF* atrial fibrillation; *CNS* obstructive sleep apnea; *OSA* obstructive sleep; *TIA* transient ischemic attack

The factors related to patients with both AF and OSA are shown in Table 2. In the univariate logistic analysis, male sex (OR = 5.13, 95% CI 2.84–9.23, *p* < 0.01), age (OR = 0.98, 95% CI 0.96–1.00, *p* = 0.01), BMI (OR = 1.18, 95% CI 1.11–1.26, *p* < 0.01), smoking (OR = 5.0, 95% CI 3.08–8.10, *p* < 0.01), major bleeding history (OR = 3.36, 95% CI 1.29–8.76, *p* = 0.01) were correlated with AF combined with OSA. Furthermore, the multivariate logistic model analysis revealed that male sex (OR = 3.02, 95% CI 1.56–5.85, *p* < 0.01), BMI (OR = 1.17, 95% CI 1.09–1.25, *p* < 0.01), smoking (OR = 2.71, 95% CI 1.58–4.68, *p* < 0.01) and major bleeding history (OR = 3.07, 95% CI 1.11–8.48, *p* = 0.03) were independent risk factors for patients with both AF and OSA. The one-year outcomes of the two groups are shown in Table 3. During 1-year follow-up, a total of 162 (8.1%), 147 (7.4%), and 15 patients (0.8%) experienced thromboembolism, stroke/TIA, and non-CNS embolism, respectively. Non-CNS embolism accounted for 9.3% of the total thromboembolisms. Compared to the patients with AF, but not OSA, the total incidence of thromboembolism in patients with both AF and OSA was numerically higher, but not significantly (10.0% vs 8.1%, *p* = 0.52). Patients with AF and OSA had similar incidences of stroke (5.7% vs 7.4%, *p* = 0.58), but a significantly higher incidences of non-CNS embolism (4.3% vs 0.6%, *p* = 0.001) than those with AF, but not OSA (Fig. 2).

**Table 2:** Univariate and multivariate logistic regression analysis of related factors of AF patients with OSA

Univariate logistic regression	OR	95% CI	*p*-value
Male	5.13	2.84–9.23	< 0.01
Age	0.98	0.96–1.00	0.01
BMI	1.18	1.11–1.26	< 0.01
Initial SBP	1.00	0.99–1.01	0.87
Initial DBP	1.01	1.00–1.03	0.06
Initial HR	1.00	0.99–1.00	0.6
*Comorbidities*			
Myocardial infarction	0.85	0.36–1.99	0.70
Coronary artery disease	1.16	0.70–1.89	0.56
Heart failure	0.89	0.55–1.45	0.65
Rheumatic heart disease	1.71	0.78–3.78	1.82
Permanent pacemaker	2.26	0.31–16.57	0.42
Hypertension	0.65	0.39–1.07	0.09
LVH by ECG or echo	0.59	0.34–1.03	0.06
Previous stroke or TIA	1.12	0.60–2.11	0.72
Smoking	5.00	3.08–8.10	< 0.01
Left ventricular systolic dysfunction	0.68	0.39–1.17	0.16
Dementia or cognitive defects	1.58	0.22–11.65	0.65
COPD	1.00	0.47–2.12	0.99
Diabetes mellitus	0.47	0.43–1.48	0.80
Hyperthyroidism	2.42	0.33–17.69	0.38
Valvular heart disease	1.08	0.56–2.08	0.82
Prior major bleeding	3.36	1.29–8.76	0.01

Multivariate logistic regression^a^	OR	95% CI	p-value
Male	3.02	1.56–5.85	< 0.01
Age	0.99	0.97–1.06	0.18
BMI	1.17	1.09–1.25	< 0.01
Smoking	2.71	1.58–4.68	< 0.01
Prior major bleeding	3.07	1.11–8.48	0.03

*BMI* body mass index; *CI* confidence interval; *COPD* chronic obstructive pulmonary disease; *DBP* diastolic blood pressure; *ECG* electrocardiogram; *HR* heart rate; *LVH* left ventricular hypertrophy; *OR* Odd ratio; *OSA* obstructive sleep apnea; *SBP* systolic blood pressure

^a^Adjusted for male, age, BMI, smoking, prior major bleeding

**Table 3:** One-year outcomes in AF patients with and without OSA

	Total	Non-OSA	OSA	*p*-value
n	1990	1920	70	
Thromboembolism	162 [8.1%]	155 [8.1%]	7 [10.0%]	0.52
Stroke/TIA	147 [7.4%]	143 [7.4%]	4 [5.7%]	0.58
Non-CNS embolism	15 [0.8%]	12 [0.6%]	3 [4.3%]	< 0.01

*CNS* central nervous system; *OSA* obstructive sleep apnea;* TIA* transient ischemic attack

**Fig. 3:**
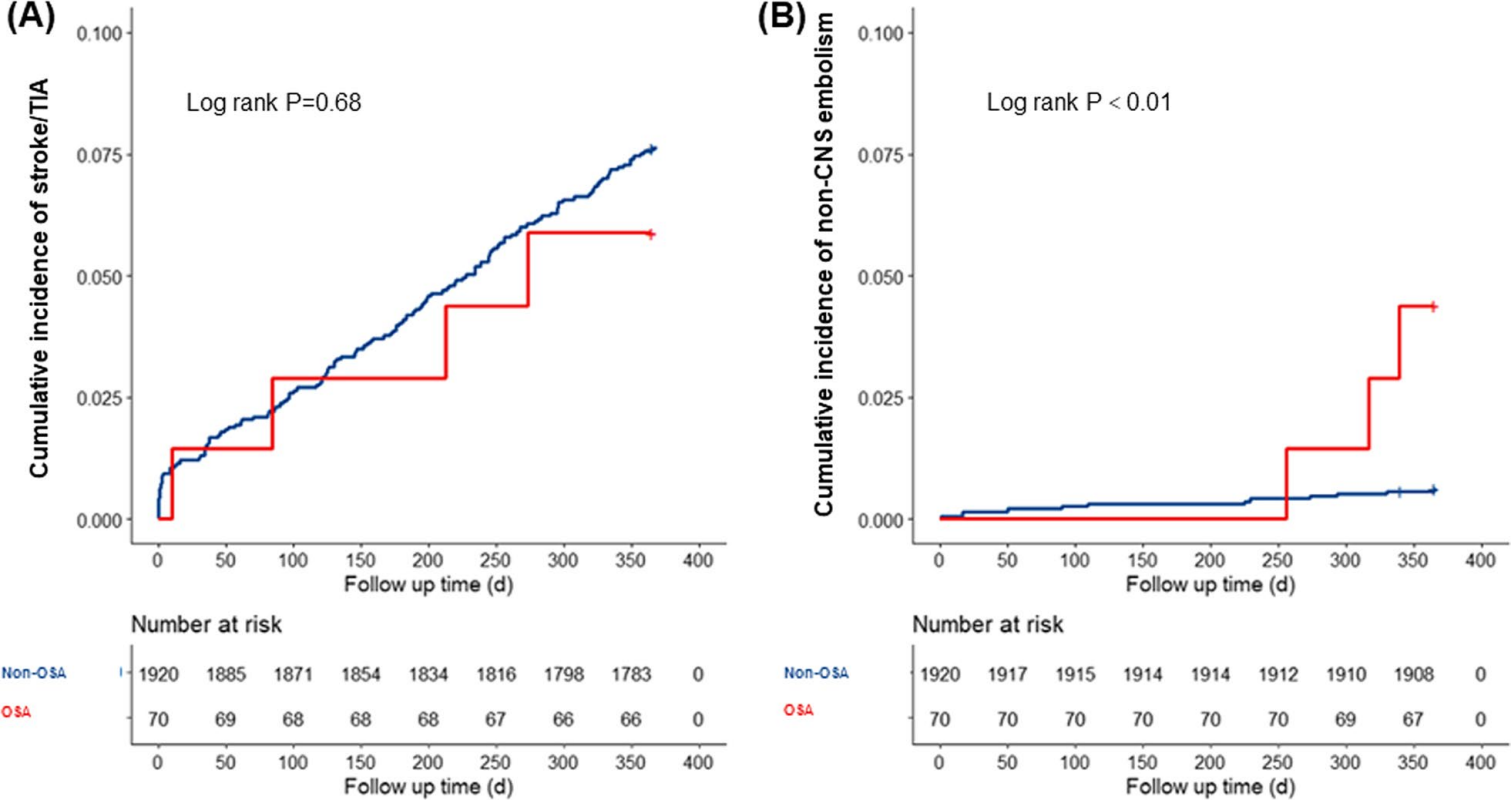
The Kaplan–Meier analysis in AF patients with and without OSA. **A** Stroke/TIA; **B** Non-CNS embolism. *AF* atrial fibrillation; *CNS* central nervous system; *OSA* obstructive sleep apnea; *TIA* transient ischemic attack

Figure 3 shows the cumulative incidence of stroke/TIA (Fig. 3A) and non-CNS embolism (Fig. 3B). Patients with AF and OSA were at a significantly higher risk of non-CNS embolism than patients with AF but without OSA (*p* < 0.01). Table 4 shows the associations between OSA and 1-year thromboembolism events in patients with AF. The univariate Cox regression analyses revealed that OSA was not correlated with the risk of stroke/TIA (HR 0.77, 95% CI 0.29–2.09, *p* = 0.61). However, OSA significantly increased the risk of non-CNS embolism (HR 6.90, 95% CI 1.95–24.45, *p* = 0.003) in patients with AF. After adjusting for additional covariates that influenced the non-CNS embolism of patients with AF, OSA was still an independent risk factor for non-CNS embolism (HR 5.42, 95% CI 1.34–22.01, *p* = 0.02).

**Table 4:** Associations between OSA and 1-year outcomes in AF patients

	HR (95%CI)	*p*-value
*Crude model*		
Stroke/TIA	0.77 (0.29–2.09)	0.61
Non-CNS embolism	6.90 (1.95–24.45)	< 0.01
*Adjusted model *^*a*^		
Stroke/TIA	0.91 (0.33–2.52)	0.86
Non-CNS embolism	5.42 (1.34–22.01)	0.02

*CI* confidence interval; *CNS* central nervous system; *HR* hazard ratio; *OSA* obstructive sleep apnea; *TIA* Transient ischemic attack

^a^Adjusted for sex, age, BMI, HR, heart failure, hypertension, diabetes mellitus, stroke/TIA, smoking, left ventricular ejection fraction < 45%

## Discussion

In the present study, we explored factors of patients with both AF and OSA and evaluated the impact of OSA on the 1-year thrombotic risk of patients with AF. We revealed that male sex, BMI, smoking, and major bleeding history were independent risk factors for patients with AF concomitant with OSA. Furthermore, this present study revealed that OSA was not correlated with the risk of stroke/TIA, but exhibited a significantly increased risk of non-CNS embolism even after multivariate adjustment.

In our study, 3.5% of patients with AF (n = 1990) from 20 emergency departments around China presented with concomitant OSA. In previous studies, the prevalence of sleep apnea in patients with AF ranged from 3.1%-62% [7–10]. The prevalence of sleep apnea varies depending on the features of the sample studied and the diagnostic criteria, thus, the exact prevalence remains unclear [11]. Although OSA is a common risk factor for cardiovascular disease, it remains underdiagnosed and undertreated in clinical practice [12]. Recently, a statement from the American Heart Association suggested that patients with recurrent atrial fibrillation after cardioversion or ablation should be screened for OSA [13]. The ESC guidelines also recommended that opportunistic screening for sleep apnea should be considered in patients with AF. However, they do not mention the clinical characteristics of sleep apnea in patients with AF who might have the greatest need of opportunistic screening [6]. Thus, it may be worth evaluating the clinical characteristics of AF with high suspicion of sleep apnea.

Among patients with both AF and OSA in the present study, 80.0% were male. Notably, after adjusting for gender, age, BMI, smoking, and major bleeding history, we found that male sex, smoking, BMI, and major bleeding history were independent risk factors for OSA in patients with AF. Consistent with our observations, previous studies also revealed that male sex, obesity, and smoking were shared risk factors for both OSA and AF [11, 14, 15]. The Wisconsin Sleep Cohort revealed that higher BMIs were associated with an increased risk of moderate-to-severe sleep apnea [14]. According to data from six representative surveys in China, the standardized mean BMI levels of the Chinese population were 24·4 kg/m2 (24.3–24.6) in 2018 [16]. The BMI of the AF with sleep apnea group in our study was markedly higher than that in the non-sleep apnea group, and even higher than the standardized mean BMI level of Chinese population. Kim et al. suggested that smoking was associated with a higher risk of OSA [17]. Esen et al. observed a higher rate of severe OSA among male smokers [18]. The mechanism of the correlation between sleep apnea and smoking may be damage and inflammation of the upper respiratory airways due to the neuromuscular protective reflexes, accompanied by cellular edema, epithelium thickening, or ciliary dysfunction due to smoking [19]. Shiao et al. explored the tendency of patients with peptic ulcer bleeding tended to have sleep apnea and stated that sleep apnea was likely an independent risk factor for peptic ulcer bleeding [20]. The underlying mechanisms may be that sleep apnea may participate in the occurrence and development of peptic ulcers and peptic ulcer bleeding through intermittent hypoxia, systemic inflammation, oxidative stress, and sympathetic activation caused by apnea episodes [20, 21]. We were the first to our knowledge to find a relationship between major bleeding history and sleep apnea in patients with AF; however, the mechanisms of this relationship cannot be determined by this analysis. Therefore, further study is needed to evaluate the internal connection. Our study suggested that sleep apnea in patients with AF shared similar risk factors with isolated sleep apnea, such as male sex, higher BMI, and smoking. Notably, patients with AF and a major bleeding history also exhibited a higher risk of sleep apnea independently.

OSA is reportedly related to arterial stiffness and hypertension [12], and increased arterial stiffness was found to be an independent risk factor for stroke [22]. Arterial stiffness is closely associated with increased systemic inflammation and endothelial dysfunction, which may lead to venous thrombosis [23, 24]. Thus, it is important to explore the relationship between sleep apnea and thromboembolism. In our study, 7.4% of the study population experienced stroke/TIA, and 0.8% of them experienced non-CNS embolism, which was similar to the incidences observed previously [25]. Yaranov et al. investigated 332 patients with AF and new OSA diagnoses and found that the incidence of stroke in the OSA group was higher than that in the AF without OSA group (25.4% vs 8.2%, P < 0.05) [26]. Dalgaard et al. also conducted a retrospective study including 22,760 AF patients from ORBIT-AF I and ORBIT-AF II and revealed that OSA was an independent risk factor for a composite endpoint of stroke and non-CNS embolism in patients with AF [27]. However, Dalgaard’s study did not separate the analysis of stroke and non-CNS embolism. Conversely, Chang et al. conducted a retrospective study that enrolled a total of 17,375 patients with diagnosed AF. Their results revealed that OSA was not an independent risk factor for stroke in patients with AF. Furthermore, when adding OSA into the CHADS_2_ score, OSA did not improve the predictive value for risk classification of AF [28]. However, the endpoints of Chang’s study did not include non-CNS embolism. In our observation, sleep apnea was found to be an independent risk factor for non-CNS embolism in AF patients, but there was no significant difference in stroke occurrence between the two groups. One potential explanation for the discrepancy may be related to atrium enlargement which is conductive to the formation of larger thrombi that may bypass the carotid orifice and cannot enter the cerebral circulation [29]. Left atrium enlargement is a common manifestation in patients with OSA [30]. Abel Romero-Corral et al. conducted a cross-sectional study of 85 patients via echocardiography and polysomnography; the study revealed that the mean left atrial volume index of patients with OSA was notably increased [31]. Another cross-sectional study from a sample of 411 old men all aged 71-years old indicated that OSA significantly correlated with left atrium enlargement [32]. Left atrium dilatation may contribute to the formation of large atrial thrombus which might be prevented from entering the cerebral circulation due to hydrodynamic, anatomic, and physical factors [29]. However, this potential mechanism is just a hypothesis, and further research is needed to verify the relationship between left atrium enlargement and non-CNS embolism in patients with OSA.

Compared with AF-related stroke, non-CNS embolism did not get enough attention due to difficulty in detecting [33]. More non-CNS embolisms may be clinically asymptomatic. However, data from four randomized clinical trials (ACTIVE-A, ACTIVE-W, AVERROES, RE-LY) of patients with AF revealed that non-CNS embolism comprised 11.5% of clinically recognized thromboembolic events in patients with AF and the mortality, morbidity and medical costs of non-CNS embolism were comparable to those of stroke [33]. The results emphasized the importance of identifying non-CNS embolism in AF patients. Our study revealed that non-CNS embolism accounted for 9.3% of the total thromboembolic events in patients with AF, which is similar to that observed in previous studies [25, 33]. According to our observations, OSA was an independent risk factor for non-CNS embolism in patients with AF. The results implied that physicians should be more cautious regarding the risk of non-CNS embolism when treating patients with AF and concomitant sleep apnea.

## Limitations

Some limitations ought to be considered in our study. First, some potential variables such as socio-economic status and nutritional conditions, which may influence the accuracy of the results, were not collected in this present study. Second, as data on the rate and method of treatment for sleep apnea were not collected, we cannot further assess the impact of continuos positive airway pressure (CPAP) therapy on thrombotic risk in patients with AF in the present study. We also did not collect echocardiographic parameters, including left atrium dimension and left ventricular ejection fraction, in this registry; thus, the influence of these variables on thromboembolism could not be evaluated. Future research is warranted to assess the association between left atrium dimension, left ventricular ejection fraction, and thromboembolism in patients with AF and OSA. Moreover, the rate of warfarin use and the anticoagulant effect were not ideal in this study because the database was completed in 2011, and the 20 subcenters included in this study represented different medical healthcare levels from rural to urban in China. However, this study was based on real-world data, indicating that efforts to improve AF management in clinical practice are still needed.

## Conclusions

The results of our study indicate that male sex, increased BMI, smoking, and major bleeding history are independent risk factors for patients with both AF and OSA. OSA was not correlated with an increased risk of stroke/TIA; however, it was an independent risk factor for non-CNS embolism in patients with AF. Our results indicated that non-CNS embolism requires focus in patients both with AF and OSA.

## Supplementary Information

**Additional file 1.** **Table S1**. INR in AF patients using warfarin.

## Data Availability

The datasets generated and analyzed during the current study are not publicly available due privacy and ethical restrictions but are available from the corresponding author on reasonable request.

## Abbreviations

AF: Atrial fibrillation
OSA: Obstructive sleep apnea
CNS: Central nervous system
BMI: Body mass index
AHI: Apnea-hypopnea index
LVEF: Left ventricular ejection fraction
TIA: Transient ischemic attack
ECG: Electrocardiography
COPD: Chronic obstructive pulmonary disease
HR: Hazard ratio
CI: Confidence interval
CPAP: Continuos positive airway pressure
