# Supplementary material for: Clinical characteristics and thrombotic risk of atrial fibrillation with obstructive sleep apnea: results from a multi-center atrial fibrillation registry study
Source: BMC Cardiovasc Disord. 2022 Jul 25;22:331. doi: 10.1186/s12872-022-02773-9 (PMC9310481; doi:10.1186/s12872-022-02773-9)
Supplement: Supplementary file 1 — Additional file 1. Table S1. INR in AF patients using warfarin. [file 12872_2022_2773_MOESM1_ESM.docx]

**Additional file 1**

**Table S1 INR in AF patients using warfarin**

|  | Total  (n=335) | Non-OSA  (n=327) | OSA  (n=8) | *p*-value |
| --- | --- | --- | --- | --- |
| INR range (n[%]) |  |  |  | 0.68 |
| ＜1.5 | 68 [20.3%] | 68 [20.8%] | 0 [0%] |  |
| 1.5 to 2.0 | 81 [24.2%] | 78 [23.9%] | 3 [37.5%] |  |
| 2.0 to 3.0 | 66 [19.7%] | 64 [19.6%] | 2 [25.0%] |  |
| ≥ 3.0 | 6 [1.8%] | 13 [4.0%] | 0 [0%] |  |

AF, atrial fibrillation; OSA, obstructive sleep apnea; INR，international normalized ratio.
